# Analysis of Pathogenic Pseudoexons Reveals Novel Mechanisms Driving Cryptic Splicing

**DOI:** 10.3389/fgene.2021.806946

**Published:** 2022-01-24

**Authors:** Niall P. Keegan, Steve D. Wilton, Sue Fletcher

**Affiliations:** ^1^ Centre for Molecular Medicine and Innovative Therapeutics, Health Futures Institute, Murdoch University, Perth, WA, Australia; ^2^ Centre for Neuromuscular and Neurological Disorders, Perron Institute for Neurological and Translational Science, The University of Western Australia, Perth, WA, Australia

**Keywords:** pseudoexons, cryptic splicing, splicing mutations, recursive splicing, poison exons, genetic disease

## Abstract

Understanding pre-mRNA splicing is crucial to accurately diagnosing and treating genetic diseases. However, mutations that alter splicing can exert highly diverse effects. Of all the known types of splicing mutations, perhaps the rarest and most difficult to predict are those that activate pseudoexons, sometimes also called cryptic exons. Unlike other splicing mutations that either destroy or redirect existing splice events, pseudoexon mutations appear to create entirely new exons within introns. Since exon definition in vertebrates requires coordinated arrangements of numerous RNA motifs, one might expect that pseudoexons would only arise when rearrangements of intronic DNA create novel exons by chance. Surprisingly, although such mutations do occur, a far more common cause of pseudoexons is deep-intronic single nucleotide variants, raising the question of why these latent exon-like tracts near the mutation sites have not already been purged from the genome by the evolutionary advantage of more efficient splicing. Possible answers may lie in deep intronic splicing processes such as recursive splicing or poison exon splicing. Because these processes utilize intronic motifs that benignly engage with the spliceosome, the regions involved may be more susceptible to exonization than other intronic regions would be. We speculated that a comprehensive study of reported pseudoexons might detect alignments with known deep intronic splice sites and could also permit the characterisation of novel pseudoexon categories. In this report, we present and analyse a catalogue of over 400 published pseudoexon splice events. In addition to confirming prior observations of the most common pseudoexon mutation types, the size of this catalogue also enabled us to suggest new categories for some of the rarer types of pseudoexon mutation. By comparing our catalogue against published datasets of non-canonical splice events, we also found that 15.7% of pseudoexons exhibit some splicing activity at one or both of their splice sites in non-mutant cells. Importantly, this included seven examples of experimentally confirmed recursive splice sites, confirming for the first time a long-suspected link between these two splicing phenomena. These findings have the potential to improve the fidelity of genetic diagnostics and reveal new targets for splice-modulating therapies.

## 1 Introduction

According to the current release of Ensembl (104.38), the 3.1 gigabases of the human genome are estimated to contain over 44,000 genes, just over 20,000 of which encode proteins (Howe et al., 2021). Based on statistics provided by Piovesan et al. (2019), protein-coding genes account for 41% of the total human genome, although exons, the transcribed segments that are retained in the mature mRNA, comprise only 4.65% of this fraction, or 1.91% of the total genome.

During and after transcription pre-mRNAs undergo splicing, a process whereby introns are excised from the pre-mRNA molecule and the ends of the flanking exons are ligated together by a multi-molecular assembly called the spliceosome. This splicing process needs to be both consistent and accurate, since an error of even a single nucleotide could render an entire transcript functionless or cause it to encode a toxic product. However, this does not mean that splicing must be perfect. Occasional splicing errors are inevitable even in healthy cells (Alexieva et al., 2021), and these aberrant transcripts are generally well-managed by error-detecting systems such as nonsense-mediated decay (NMD) (Hug et al., 2016)—but never without some energy cost to the cell.

There is, therefore, an ancient and relentless evolutionary pressure on all eukaryotes to splice pre-mRNAs as efficiently as possible. This raises the question of why evolution allows introns to persist in the first place. Much effort has been devoted to investigating this question, and there appears to be no single answer (Chorev and Carmel, 2012; Jo and Choi, 2015). Stated briefly, introns contain numerous regulatory elements that enable alternative splicing and fine control of gene expression, and the presence of these elements may have indirectly modulated the efficiency of natural selection for eukaryotic life on Earth. Recent research also reveals a regulatory role for conserved exon-like sequence elements within some introns, such as poison exons, decoy exons and recursive splice sites (Conboy, 2021). However, this does not imply that every nucleotide of every intron is of equal importance as a nucleotide of coding sequence. Pathogenic mutations are discovered within known coding regions at a rate of at least 25% (Sawyer et al., 2016), much higher than the exonic proportion of genes (4.65%), indicating that mutations in introns are generally better tolerated.

Pathogenic mutations within introns are frequently found to affect splicing. Splicing mutations account for about 9% of all identified pathogenic mutations, though this figure also includes exonic mutations with splicing impact (Stenson et al., 2017). Most pathogenic splicing mutations weaken the definition of conserved branch point, acceptor site or donor site motifs of canonical exons, resulting in skipping of whole or partial exons, or inclusion of partial or whole introns (Abramowicz and Gos 2018). In some cases, however, a mutation will cause part of an intron to be erroneously spliced into mature transcripts as if it were an exon. These inclusions, when they occur, are called pseudoexons (PEs) or cryptic exons.

Mutations that cause pathogenic PEs in germline cells could accurately be described as ‘rare but ubiquitous.’ They are rare in that they appear to account for very few unique splicing events, yet they are also ubiquitous in the sense that they can potentially arise in any gene with at least one intron, and do not exhibit any noticeable bias towards particular genes or cell types. Consequently, any new insights into how and why pathogenic PEs arise may have implications for numerous genetic diseases.

Surprisingly, there have been very few focused studies on the general characteristics of pseudoexons. Královičová and Vořechovský (2007) examined the exonic splice enhancer and silencer (ESE and ESS) content of pathogenic PEs and found that they were intermediate between introns and canonical exons. A 2010 study by Vořechovský investigated the correlation of PEs with transposable elements and found that MIRs (Mammalian-wide interspersed repeats) and antisense *Alu* elements were statistically overrepresented in pseudoexon sequences, which they attributed to exon-like characteristics that these elements naturally possess. Dhir and Buratti (2010) directly examined the mutations that activate PEs and proposed that they could be divided into five distinct categories: splice site or branch point creation, ESE gain or ESS loss, internal deletion, intragenic inversion, and loss of a flanking canonical splice site. Subsequent observations by Romano et al. (2013) of PEs in cancer-associated genes implicitly supported Dhir and Buratti’s categories, although Romano et al., additionally noted the possibility of PEs arising through other, as-yet uncharacterised mechanisms. More recently, Vaz-Drago et al. (2017) collated additional examples of PEs fitting Dhir and Buratti’s first two categories and observed a distribution of PE sizes similar to that of internal canonical exons. Lastly, our own review of pseudoexons in the *DMD* gene (Keegan 2020) examined the coincidence of PEs with reported recursive splicing, and suggested mutation-driven exonization of recursive splice sites as an explanatory mechanism for some PEs.

Although this handful of reviews provided many useful insights into the mechanisms of PE pathogenesis, all of them (including our own) were limited by their small sample sizes. For the older reviews in particular, this impediment was largely attributable to the scarcity of pseudoexon reports available at the time they were written, although a lack of clarity in how some primary reports presented their pseudoexon data may also have contributed. In recent years, the rate of reports of new pseudoexons has continued to accelerate as new technologies make RNA analysis faster, cheaper and more accurate. Therefore, it is timely to undertake a new and comprehensive analysis of pseudoexons and their instigating mutations.

In this report, we present a catalogue of 413 germline pseudoexon variants, which were as many as we could find through a thorough search of the literature. To our knowledge, this is the first time a PE dataset of this size has been assembled. Our analysis of this data discovered that 15.7% of PEs exhibit splicing activity at one or both of their splice sites in non-mutant cells, suggesting that many reported PEs might be more accurately reclassified as mutant variants of intronic splice regulating elements within introns, such as poison exons or decoy exons. Importantly, these shared intronic splice sites include seven empirically-verified recursive splice sites, confirming for the first time a long-suspected link between these two splicing phenomena.

Additionally, while our examination of the mutations that cause PEs largely supported the observations of Dhir and Buratti (2010), the expanded sample size of our catalogue allowed us to suggest refinements and additions to their original five categories.

## 2 Materials and Methods

Although PEs have been observed in a highly diverse range of genes and cell types, they remain a relatively rare splicing phenomenon, and it was therefore unavoidable that any analysis of their characteristics would require some degree of compromise between specificity and sample size. In this section we will outline the criteria we adopted for determining what data to include in our analyses, what data to exclude, and why.

### 2.1 Working Definition of “Pseudoexon”

Our intention for this report was not to analyse all forms of cryptic splicing, but specifically those instances where splicing of non-canonical exons in a gene increased as the result of pathogenic mutations in that gene.

In a previous report on *DMD* gene PEs (Keegan, 2020) we suggested the following definition for PEs:

*“[A pseudoexon is] any continuous tract of a transcribed gene that: 1) does not overlap, adjoin or duplicate any sense-strand sequence of that gene’s canonical exons; 2) bears an acceptor splice site motif at its 5′ end and a donor splice site motif at its 3′ end; and, 3) via both these motifs, is spliced into a measurable proportion of the mature transcripts of that gene in at least one proband.*”

We adopted a streamlined version of that definition for this report:

*A germline pseudoexon is any continuous tract of a transcribed gene that: 1) does not overlap, adjoin, or duplicate any sense-strand sequence of a canonical exon; and 2) is spliced into mature transcripts of that gene in non-cancer cells of at least one proband 3) partly or wholly due to mutation in that gene.*

This modified definition allowed for the inclusion of PEs spliced via non-canonical motifs and PEs spliced in first-exon or last-exon orientations. It also enforces the exclusion of canonical exons introduced into other genes *via* gene fusions, which could technically have been classed as PEs under the previous phrasing.

#### 2.1.1 Exclusion of Cancer Pseudoexons

While many reports have detailed the correlation of PEs and various forms of cancer, we chose to limit our analysis to only those PEs that arose from germline mutations. This was because cancer cells often exhibit idiosyncratic changes in splice factor expression, and a general relaxation of splicing stringency, in comparison to non-cancer cells (Zhang et al., 2021). As such, we judged it would not be valid to make like-for-like comparisons between PEs in cancers and those in germline cells. However, we did include PEs arising from germline mutations in cancer-associated genes, such as *NF1* and *ATM*, as in these cases carcinogenesis appeared to be a result of PE inclusion rather than the cause of it.

#### 2.1.2 Exclusion of Pseudoexons in Non-Humans

Although PEs have been observed in other animal species (e.g., Smith et al., 2007; Gómez-Grau et al., 2017), such observations are even rarer than they are in humans, a disparity that probably stems from the lower level of interest in mutation analysis in non-human species. We determined that the inclusion of PEs from non-human species would only offer a modest increase to our study’s sample size at the cost of greatly generalising its conclusions, and therefore limited its scope to human PEs only.

#### 2.1.3 Inclusion of Pseudoexons Identified *via* Transfected Minigene/Midigene Constructs

Wherever possible, it is ideal for investigations of splicing mutations to use RNA from patient cells that natively express the gene of interest. Unfortunately, this is not a practical option for many genes. For example, the Stargardt disease gene *ABCA4* is primarily expressed in kidney and retinal cells, and it is rare that a biopsy of either of these internal tissues can be justified. Instead, many researchers utilise HEK293T cells (immortalized human embryonic kidney cells), which they transform with minigene constructs of the *ABCA4* region of interest to model the effects of the mutation. In other cases, transformed COS cells (immortalised simian kidney fibroblast-like cells) or transformed HeLa cells (immortalised cervical cancer cell line) are used for similar purposes.

We elected to include in our dataset most of the PEs identified via minigene constructs, provided that the minigene constructs contained, at minimum, the entire intron surrounding the PE and both the flanking canonical exons. This minimised the chance of including a PE produced by construct-specific changes to its proximal sequence elements instead of the patient’s mutation. For the same reason, we also decided that if the effects of a splicing mutation were observed in both modified and unmodified cells, only the observations from the unmodified cells would be considered.

#### 2.1.4 Inclusion of ‘Terminal-Pseudoexons’

Virtually all of the reports surveyed in this analysis described internal PEs, i.e., PEs that were spliced into a gene transcript somewhere between its canonical first and last exons. However, there were some reports that appeared to detail “terminal-pseudoexons” (tPEs) arising from mutations that caused non-canonical sequence inclusions at the 3′ ends of largely canonical transcripts. We determined that these putative tPEs would only be classed as such, and included in our catalogue, if they could meet three criteria:

(1) They possessed a novel acceptor site.
(2) They did not overlap or adjoin any sense-oriented canonical exon sequence.
(3) They possessed a functional polyadenylation site.

This third criteria is perhaps the most important, as it distinguishes “true” tPEs from more common events such as incomplete splicing and/or partial intron inclusion, which typically result in rapid nonsense-mediated decay of the affected transcript. We therefore only included tPEs if the supporting RNA analysis directly confirmed a *de novo* polyadenylation site, either *via* 3′ RACE or through whole transcript sequencing.

### 2.2 Quality and Method of RNA Sequencing

The primary sources collated in this report span nearly 40 years of genetics research. As such, the RNA sequencing methods used by these sources run the full gamut of technologies, from S1 nuclease mapping to Nanopore. With few exceptions, we were agnostic towards the RNA sequencing technology used, provided that the PE sequence and splice sites could be mapped to the genomic reference sequence with a high degree of certainty. The level of detail provided in most reports made this a straightforward process, especially for those that had included Sanger sequence traces of the PE splice site junctions or Varnomen-format descriptions matched to specific reference sequences. In other cases, it was necessary to deduce PE sequences from precise but indirect details, such as the stated length of the PE relative to its instigating mutation.

In some reports, the stated boundaries of one or more PEs appeared to be exceptions to the U2-type GY-AG splicing that predominates in the dataset and did not fit the established motifs of U12-type splice sites either (Turunen et al., 2013). Given the rarity of such non-canonical exon boundaries in the human transcriptome (Parada et al., 2014), we judged that it was appropriate to exercise additional scrutiny, and we only included non-canonically spliced PEs if their supporting sequence data was unequivocal.

In cases where the published detail of a PE report was insufficient to determine the PE sequence, we contacted the corresponding authors with requests for further detail and have cited those that graciously responded as “Pers Comms” where appropriate.

### 2.3 Quantity of Pseudoexon Inclusion in Mature Transcripts

Our dataset does not incorporate quantitative data for the frequency of inclusion of each PE. While it could be argued that this weakens the analyses in some respects—since PEs with 10% inclusion are treated identically to PEs with 100% inclusion—we reasoned that avoiding quantitative analysis would be the “lesser of two evils.” Collectively, this study’s source reports show enormous variation in the genes studied, the types of cells used, and the methods of RNA analysis, with many producing sequence data that was non-quantitative or at best semi-quantitative. Accurate and objective standardisation of these data would have been all but impossible and risked generating misleading conclusions.

### 2.4 Search Criteria

Literature search was performed using Google Scholar and the Murdoch University academic research portal *FindIt*. Individual searches of the following terms were conducted through both portals: “*pseudoexon*,” “*pseudo exon*,” “*cryptic exon*,” and “*deep intronic*.” The first three of these terms comprise the most common descriptors for PEs, while the fourth term served as a “safety net” to return any publications of deep intronic splicing mutations that may have reported on PEs using unexpected terminology.

In addition to scrutinising each paper for useful data, we also performed searches within each paper for the key terms mentioned above and investigated any references that were cited against these mentions. This allowed us to discover additional PE reports that, for various reasons, had escaped capture by our direct searches.

Similarly, we are indebted to the authors of several previous reviews of PEs, whose works led us to additional primary sources that had eluded discovery through the above methods (Královičová and Vořechovský, 2007; Dhir and Buratti 2010; Vořechovský 2010; Romano et al., 2013; Vaz-Drago et al., 2017). We have also incorporated data for *DMD* PEs that was originally collated for a previous report (Keegan 2020).

### 2.5 Construction of Pseudoexon Catalogue

#### 2.5.1 Transcribed Pseudoexon Features

The rarity of PEs, coupled with broad variability in how they were reported, made it unfeasible to automate their annotation. Therefore, all annotation of PE data was performed manually by the authors. This approach was further justified *post hoc* by discoveries of minor inconsistencies in numerous PE reports (e.g., stated splice sites or lengths that differed from those shown in the published figures), errors that would have escaped detection by any automated process and led to inclusion of inaccurate data.

For each PE, annotated data fields included the name of the affected gene using current nomenclature, as listed on the Genecards Human Gene Database (Stelzer et al., 2016); the sequence of the PE; the sequences of the flanking exons to which it was spliced; the cell or tissue type(s) in which its splicing was observed; the Varnomen cDNA code for the instigating mutation(s) if present and known (den Dunnen et al., 2016); additional notes if relevant; and citations for the primary sources of the data.

While transcript reference sequences are included for the encompassing gene of each PE, in many cases a specific refseq ID was not explicitly declared in the source. The listed reference sequences instead correspond to the lowest-numbered transcript variant (usually TV1) that matched the splicing patterns observed and have been included to allow the use of cDNA-type Varnomen mutation codes, which are more human-readable than genomic codes. Chromosomal coordinates and all other genomic features refer to the most recent human genome assembly, GRCh38.p13.

#### 2.5.2 Intron Numbering

Historically, unique identifiers have been employed when referring to the introns of certain genes, such as *NF1* and *CLRN*. For consistency, we have ignored these in favour of simple 1-to-*n* numbering for all transcript variants, but caution the reader that this may create the appearance of discrepancies when referring to some cited reports.

#### 2.5.3 Assignment of Unique Pseudoexon IDs

Each pseudoexon was assigned a unique ID according to the name of its gene, the number of the encompassing intron, and an alphanumeric identifier according to its similarity to other pseudoexons in that intron. For example, two pseudoexons with completely distinct sequence were reported in the 17th intron of the *ATM* gene and were IDed as *ATM*-17-1 and *ATM*-17-2, while the two PEs reported in *ATM* intron 27 were IDed as *ATM*-27-1a and *ATM*-27-1b due to sharing an acceptor site.

#### 2.5.4 Citations

While descriptions of most PEs were limited to a single report, some were reported multiple times by different research groups and to varying levels of detail. An extreme example of this is *CFTR-*7-1, a prevalent disease allele for cystic fibrosis that has been studied extensively. In the interest of clarity, we limited our citations for each PE to its earliest known report, including later reports only if they substantially added to the characterisation or were already cited for unique observations of other PEs.

#### 2.5.5 Derived Features

Maximum entropy (MaxEnt) splice site scores were calculated via the Burge Laboratory’s *MaxEntScan* web-tool (Yeo and Burge, 2004). Sizes and distances were directly calculated using spreadsheet formulae.

## 3 Pseudoexon Mutation Characteristics

### 3.1 Pseudoexon Splice Motif Mutations

#### 3.1.1 Pseudoexon Donor Motif Mutations

**FIGURE 1:**
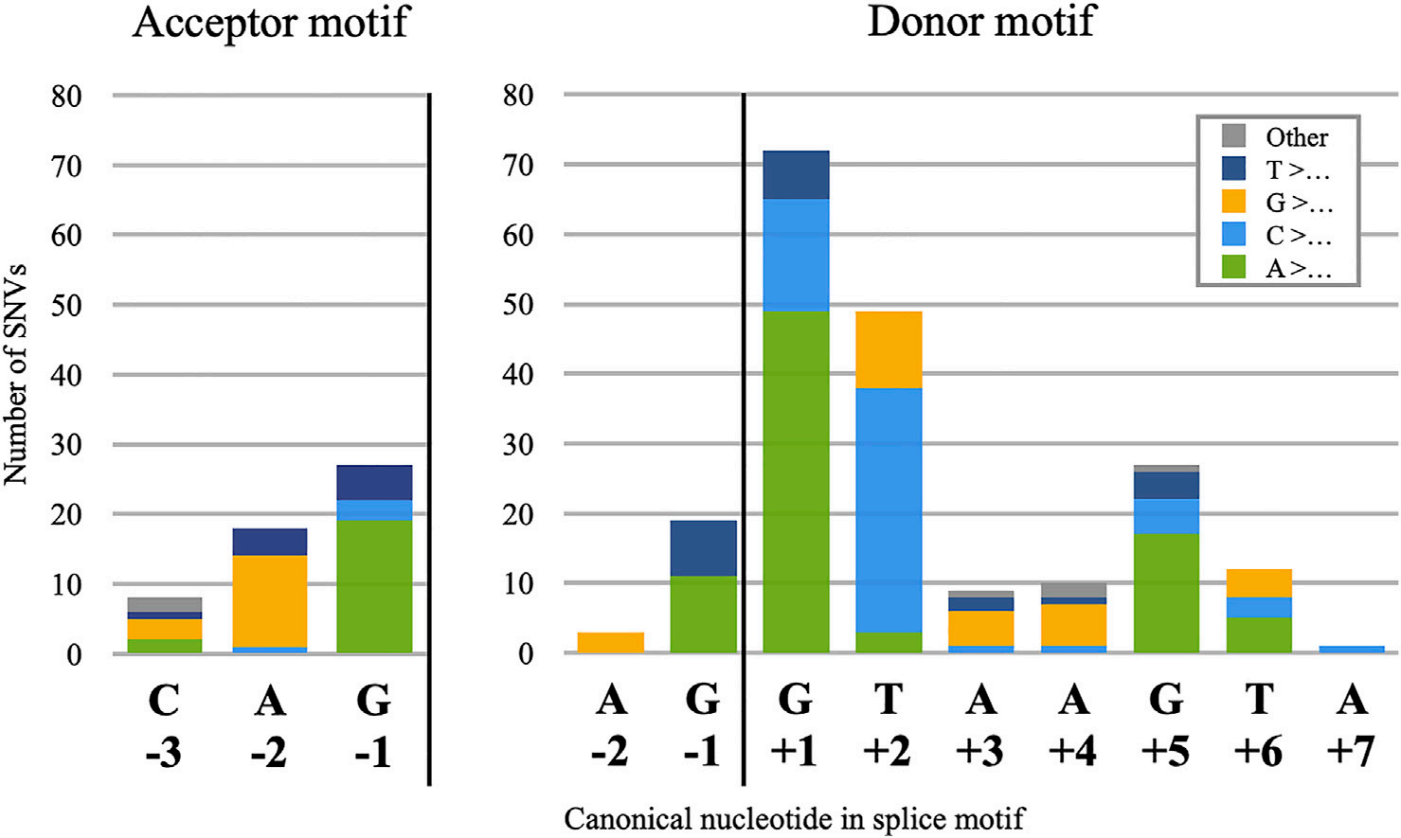
Positional frequencies of 255 pseudoexon splice-motif SNVs. Stacked columns are categorised by the identity of the reference nucleotides, which in 249 of the 255 cases were mutated to the most frequently observed nucleotide at that position of the motifs, as shown on the *X*-axis. Exceptions (6) are categorised as “Other.”

Donor splice site mutations are the most frequently observed cause of PE pathogenesis, comprising 210 of the 359 catalogued distinct mutations. Of these 210, 202 are single nucleotide variants (SNVs), and the frequency of mutation for each nucleotide position relative to the donor site (Figure 1, right) approximates the degree of nucleotide conservation observed for that position in canonical sites (Ma et al., 2015). This may be a logical consequence of how the spliceosome binds to candidate donor sites: because the effect of nucleotide identity on spliceosome binding varies greatly across the motif, changes at the most essential positions will have the greatest effect and the best chance of “breaking through” the silencing mechanisms that would otherwise prevent detectable levels of splicing. This is corroborated by our observation that no PE in our dataset was instigated by an SNV at the donor site −3 position, despite this nucleotide falling inside the donor site motif. Because the −3 position is not highly conserved, any change in this nucleotide is unlikely to cause a noticeably pathogenic increase in PE inclusion.

However, we did note a single PE with a C>A SNV 7 nt 3′ of the donor splice site (*MMUT*-11-1b). Although the donor site motif is traditionally considered to end at the 6th 3′ nucleotide, a comprehensive analysis of human splice sites (Ma et al., 2015) reveals a substantial bias towards purines at the +7 position (59% A/G). As this *MMUT* SNV is a pyrimidine-to-purine transition, the simplest explanation of it is as a donor site mutation.

#### 3.1.2 Pseudoexon Acceptor Motif Mutations

Acceptor splice-site mutations account for 53 of the catalogued PE mutations, and all but one of those (*DMD-*30-1) were SNVs. Despite the greater size of the acceptor motif compared to the donor, pathogenic acceptor site SNVs were limited almost entirely to positions −2 and −1, with only an additional eight at −3 (Figure 1, left). As with the distribution of donor site mutations, this appears to be a result of low conservation at positions +1 to +3 and the low impact of individual nucleotides in the −20 to −4 range, with pathogenic mutations in this latter region apparently impacting branch point definition more than they did acceptor site definition. We also noted a single case of an SNV at the PE acceptor +3 position, (*ABRAXAS1*-5-1) but chose to analyse this as an internal mutation since its effect on the acceptor splice score was negligible.

Our observations of position frequency in PE donor and acceptor splice motif SNVs generally accorded with those of Krawczak et al. (2007), who examined a much larger set of gain-of-function splice mutations; and with those of Sakaguchi and Suyama (2021), who examined a smaller dataset consisting entirely of novel PEs. We also noted that the number of transition SNVs—purine-to-purine or pyrimidine-to-pyrimidine nucleotide changes—was approximately double that of transversion mutations, at 165 and 92, respectively. This accords with prior observations of how often each mutation type is generally observed in the human genome (Jiang and Zhao 2006).

### 3.2 Pseudoexon Internal Mutations

We catalogued 37 examples of PEs caused by sequence changes between the PE splice site motifs. In five of these examples (*COL4A5*-37-1, *DMD*-11-2, *DMD*-34-1, *DMD*-48-1 and *GNAS-AS1*-4-1—see Supplementary Table S1) the mutation was a >10 kb deletion that brought a latent acceptor-donor motif pair into conjunction. In these cases, we assumed that sheer distance between the splice sites was the chief silencing element that had been lost and did not analyse these further. Of the remaining 32 cases, there was one PE (*GLA*-4-1) caused by a 113 nt insertion, three PEs caused by 2–4 nt deletions, and 28 PEs caused by SNVs (Supplementary Table S2).

The positions and predicted effects of the 31 unique mutations showed much greater variability than the mutations affecting PE splice motifs. Although we could not distinguish any obvious patterns in their locations within the PEs, we noted that the primary reports consistently described these mutations as gains of ESE motifs and/or losses of ESS motifs within the PEs. Most of these assessments were made *via* an assortment of RNA motif analysis utilities, some of which are no longer available. We therefore standardised our re-analysis to a single utility, *HExoSplice*, which was designed specifically for analysing this type of mutation (Ke et al., 2011; Tubeuf et al., 2020). Because *HExoSplice* only calculates scores for SNVs, we derived scores for the deletion and insertion mutations manually by subtracting the total score for the wild-type exon from the total score of the mutant. Impressively, *HExoSplice* correctly predicted the directionality of 29 out of the 31 mutations with a net increase in the score (∆Hx). There were no obvious similarities between the two SNVs with negative ∆Hx scores (*ABCA4*-30-1c and *DMD*-32-1b). We suggest that in both these cases, the mutations may have altered binding of splicing factors specific to those genes or cells, as this kind of specific effect cannot be accurately predicted by a generalised tool such as *HExoSplice.*

### 3.3 Pseudoexon Branch Point Mutations

**FIGURE 2:**
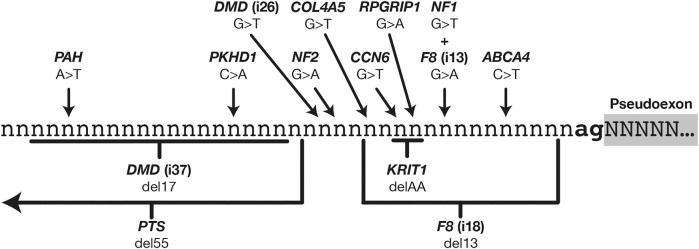
Relative locations of 14 mutations that instigate pseudoexons *via* enhancement or creation of branch point motifs.

Just 14 of the 359 PE mutations were ultimately classified as altering pseudoexon branch point definition, and these mutations showed considerable variation in their nature and location relative to the PE acceptor site (Figure 2). Historically, branch point mutations have been poorly understood and have proven more challenging to predict than other splicing mutations (Canson et al., 2020). Despite this variability we observed that the mutations we had catalogued were remarkably consistent in their effects on branch point characteristics (Table 1).

**TABLE 1:** Pseudoexon mutations that enhance pseudoexon branch point motifs.

Gene	Intron	#	Mutation	AGEZ	Max BPS	Pyr SNV?	References
*ABCA4* (NM_000350.3)	6	1	c.769-784C>T	17	0.22	**Yes**	Sangermano et al. (2019)
*CCN6* (NM_003880.4)	2	1	c.49-763G>T	24	−0.15	**Yes**	Garcia-Segarra et al. (2012)
*COL4A5* (NM_000495.5)	29	1	c.2395+1275C>G; c.2395+1292G>T	**13 -> 59**	**−1.28 -> −0.03**	**Yes**	Wang et al. (2021)
*DMD* (NM_004006.3)	26	2	c.3603+820G>T	**16 -> 42**	**0.57 -> 1.00**	**Yes**	Waddell et al. (2021)
	37	2	c.5325+1740_5325+1757del	17	**−1.33 -> −0.06 ** **(MC)**	—	Bovolenta et al. (2008)
*F8* (NM_000132.4)	13	2a	c.2113+461_2113+473del	74 -> 61	**0.83** **(MC)**	—	Jourdy et al. (2018)
	18	2b	c.5999-798G>A	**8 -> 65**	0.23	No	Pezeshkpoor et al. (2013)
*KRIT1* (NM_194456.1)	6	1	c.262+132_262+133del	**9 -> 88**	0.14	—	Riant et al. (2014)
*NF1* (NM_001042492.3)	8	3	c.889-941G>T	**8 -> 21**	−0.73	**Yes**	Pros et al. (2008)
*NF2* (NM_000268.4)	5	1	c.516+232G>A	**15 -> 40**	**0.06 -> 3.37**	No	De Klein et al. (1998)
*PAH* (NM_000277.3)	11	1	c.1199+502A>T	34	**−1.64 -> −0.16**	Yes	Jin et al. (2021)
*PKHD1* (NM_138694.4)	56	1	c.8798-459C>A	46	**−1.42 -> 1.95**	No	Chen et al. (2019)
*PTS* (NM_000317.3)	2	1b	c.163+696del55	**17 -> 28**	**−1.83 -> 1.01 ** **(MC)**	—	Meili et al. (2009)
*RPGRIP1* (NM_020366.4)	12	1a	c.1468-263G>C	**10 -> 24**	−0.56	**Yes**	Jamshidi et al. (2019), Zou et al. (2021)

“AGEZ,” “Max BPS,” and “Pyr SNV?” changes that are predicted to enhance branch point definition are in bold text. Cases where deletion mutations moved a branch point site closer to the AGEZ/acceptor site are noted in the “Max BPS” column as “(MC).”

Branch point definition requires two elements: a functional branch point site (the motif for which is weakly conserved in U2 introns) and a continuous AG-exclusion zone (AGEZ) connecting the branch point to a downstream acceptor splice site. In 12 out of the 14 cases shown here, the effect of the mutation was to “close the circuit” between an acceptor site and a branch point, through some combination of improving an in-range branch site motif (as predicted by *SVM-BPfinder* with “AGEZ only” selected—see Corvelo et al., 2010), increasing the size of the AGEZ, or moving an existing branch point closer to an AGEZ (Gooding et al., 2006).

Two exceptions to this rule were *ABCA4*-6-1 and *CCN6*-2-1, both instigated by SNVs that modestly increased their acceptor site scores (6.63 -> 7.12 and 3.12 -> 5.43, respectively) but did not affect their AGEZ size or branch point scores. Although we could defensibly have classified these as acceptor-motif mutations, we noted that every other acceptor-motif mutation fell within three nucleotides 5′ of the PE (Figure 1, left) and would therefore directly affect binding of the U2AF35 spliceosome component (Voith von Voithenberg et al., 2016). We reasoned that the positioning of the *ABCA4*-6-1 and *CCN6*-2-1 mutations within their respective polypyrimidine tracts suggested at least some interaction with other spliceosome components. A third mutation similar to these (c.639+861C>T) was reported in *GLA* (Filoni et al., 2008), although the affected exon in this case was subsequently classified as a canonical exon of transcript variant NR_164783 (Supplementary Table S3). A causative mechanism was not empirically confirmed for any of these three mutations. However, a fourth mutation, similar in type but opposite in effect, was reported by van der Wal et al. (2017). This mutation (c.-32-13T > G) occurred 13 nt 5′ of exon 2 in the gene *GAA* and caused skipping of all or part of exon 2. In this case, Van der Wal et al., were able to empirically demonstrate that the splicing disruption arose from a loss of U2AF65 binding at the mutation site.

The U2AF65 protein is a U2 spliceosome component that binds pyrimidine tracts 5′ of exon acceptor sites, interacting with the U2 snRNP to facilitate branch point recognition (Valcárcel et al., 1996). It has multiple known pyrimidine-rich binding motifs (Paz et al., 2014; Drewe-Boss et al., 2018). Since the *ABCA4*-6-1 and *CCN6*-2-1 mutations are both SNVs that create new thymine nucleotides, we theorised that the true pathogenic effect of these mutations is creation or enhancement of a U2AF65 binding site. Unfortunately, we could not test this prediction as there is at present no *in silico* tool for predicting U2AF65 binding that incorporates all known motifs. We therefore added “new pyrimidine nucleotide” as a crude predictor of U2AF65 binding.

We also noted that these three mutations are predicted by *Splice Aid 2* (Piva et al., 2012) to create new PTBP1 (hnRNP I) binding sites. Although PTBP1 plays a complex role in splicing (Lou et al., 1999; Han et al., 2014) it is generally observed to silence nearby exons. However, in some cases PTBP1 has been shown to indirectly enhance 3′ exon inclusion by antagonising splicing repressors (Paradis et al., 2007) or preventing erroneous binding of U2AF65 (Sutandy et al., 2018), so we cannot rule out a possible mechanistic role for PTBP1 in these three mutations. There is clearly a need for an accurate and comprehensive *in silico* tool that can predict the effects of all types of branch point mutations, as they remain something of a blind spot in splice mutation research.

### 3.4 Distal Mutations

All the pathogenic mutations discussed thus far have entailed some form of direct improvement to the exon-like characteristics of the PE, be it the splice sites, branch point or local enhancer/silencer balance. However, we have also catalogued 35 cases of PEs being instigated by intragenic mutations outside the span of the PE’s branch point and donor site motif, sometimes multiple canonical exons and tens of kilobases away. When viewed individually, the aetiology of these PEs may appear baffling, but examining them *en masse* reveals many important common features.

#### 3.4.1 Decreased Definition in Adjacent Canonical Exons

In their 2010 report, Dhir and Buratti proposed that loss of a canonical splice site “facing” a pseudoexon—i.e., a 5′ donor site or a 3′ acceptor site–may be a general mechanism of pseudoexon pathogenesis. Impressively, although their prediction was based on just five supporting examples (*BRCA2-20-*1a, *CFTR-*3-1, *IDS-*3-1a, *IDS-*3-1d and *MMUT-*11-1d) we found that it was generally supported by the characteristics of 13 of the additional mutations we collated (Supplementary Figure S1), albeit with refinements to the original terms of their category.

The ten examples shown in Supplementary Figure S1A most closely fit the terms of Dhir and Buratti’s original category, as all entailed loss or weakening of a 5′ donor or 3′ acceptor site. This includes one mutation that appeared to weaken a 3′ branch point (*GAA*-1-1), thereby indirectly weakening definition of the acceptor site, and one case (*DGKE*-5-1) where a mutation created a cryptic upstream donor site but left the original intact, indirectly weakening its definition via competitive effects. In addition, we also observed three examples of mutations that induced skipping of a whole exon (Supplementary Figure S1B)—including an alternative transcript variant caused by the same *GAA*-1-1 branch point mutation shown in Supplementary Figure S1A—and three examples of mutations that caused a net loss of enhancers within a flanking canonical exon (Supplementary Figure S1C), as indicated by their negative ∆Hx scores. We were also interested to note that in every case where a PE was spliced to an alternative flanking splice site—whether through cryptic splice site activation or whole exon skipping - the new site always had a lower MaxEnt score than the unmutated original.

However, this pattern is further complicated by three cases of PEs instigated by mutations in “next-but-one” neighbour exons that were not directly spliced to the PE (Supplementary Figure S1D). These cases may be a consequence of the exons near these PEs having tightly linked splicing fates, e.g., a loss of definition in *MMUT* exon 4 exerts a similar effect as a hypothetical loss of definition in exon 5 would. An analogous example of this kind of splicing effect can be seen in cases where a mutation within a single canonical exon caused skipping of both that exon and adjacent, unmutated exons (Fisher et al., 1993; Suzuki et al., 2016). We also note that there is substantial evidence of exons in many genes being consistently spliced out of transcription order (Takahara et al., 2002; Attanasio et al., 2003; Kim et al., 2017) and speculate that this, too, may contribute to the aetiology of some highly distal pseudoexon mutations. In general, exon-like tracts in late-spliced introns could be more vulnerable to the knock-on effects of splicing changes elsewhere in the maturing transcript, while those in early-spliced introns remain insulated by sheer distance.

We also observed that one of the three mutations shown in Supplementary Figure S1D (*DMD*-3-1a) was an intra-exon mutation with a positive ∆Hx score, indicating an increase in the definition strength of that exon. Although this may represent a valid counterexample to the prevailing trend of pathogenically decreased exon definition, we tentatively dismissed it as another of the inevitable minority of incorrect predictions made by the *HExoSplice* algorithm and note that one of the two other such examples we observed also occurred in the *DMD* gene (*DMD*-32-1b—see Supplementary Table S2).

Considered in aggregate, these observations suggest the common mechanism underlying these PEs is a comparative weakening of definition in the successfully spliced neighbouring exons; and *vice versa,* that the definition strength of exons flanking an intron can be an important mechanism for silencing latent PEs within. This hypothesis is supported by prior observations of stronger splice motifs in exons flanking large introns (Farlow et al., 2012), since larger introns could generally be assumed to have a higher chance of containing at least one latent PE. The infrequency with which these types of PEs arise, compared to PEs generated by direct enhancement of their donor or acceptor splice sites, may be a result of general selective pressure against splice-competent elements within intron tracts. We also speculate that pseudoexons of the kind seen in Supplementary Figure S1C may be more common than suspected and could account for the pathogenic nature of some synonymous variants (Shi et al., 2019), perhaps having escaped detection in prior experiments due to nonsense-mediated decay (NMD) of the affected transcripts.

#### 3.4.2 Novel Pseudoexon Mutation Categories

We observed 17 examples of PE mutations that bore no resemblance to Dhir and Buratti’s five categories, some of which bore sufficient similarities to each other to justify new categories (Supplementary Figure S2).

##### Proximity to a Directly Mutated Pseudoexon in the Same Intron

The first of these categories comprises four cases where PEs were apparently instigated by the activation of a second PE in the same intron (Supplementary Figure S2A). In all four cases the mutation in the ‘primary’ PE created or enhanced a donor site, and all occurred in introns of similar sizes, ranging from 1,212 nt (*MYBPC3* intron 20) to 2,582 nt (*MYBPC3* intron 12). It is also notable that in each of these cases, the primary PE introduces a flanking splice site as strong or stronger than that of the nearby canonical exon. For example, the primary PE in *F8-*12-2a introduces a downstream acceptor site stronger (MaxEnt = 8.80) than that of exon 19 (MaxEnt = 6.91). This would appear to contradict the pattern of PEs arising from weakening of flanking exon definition. However, it may be that in these cases the intron subdivision caused by splicing of the primary PEs leads to a disruption of splicing co-ordination (Drexler et al., 2020), and a consequent loss of silencing of the secondary PE that exceeds the expected gain of silencing from the primary PEs’ strong flanking splice sites. An assay of intron splicing order in cells carrying these mutations, or other similar mutations, may shed much light on the processes involved.

##### Loss of Upstream Polyadenylation Motifs

There was a single case of a PE arising from an intragenic region due to an upstream deletion (*EPCAM*-7-1, Supplementary Figure S2B). While we would otherwise hesitate to define an entire category by a single exemplar, in this case the causative mechanism is straightforward enough to justify it: Genomic deletion of the latter two exons of *EPCAM,* which necessarily entailed deletion of that gene’s polyadenylation signals, permitted transcription to continue through the intergenic region and into the 3′ gene *MSH2*, which shares *EPCAM*’s sense-strand orientation. By chance, this novel “intergenic intron” contained a 111 nt tract that was sufficiently exon-like to be spliced to the neighbouring canonical internal exons, namely *EPCAM* exon 7 and *MSH2* exon 2 (Ligtenberg et al., 2009).

Although this is the only example of this type of PE that we catalogued, similar PEs could occur in other cases where a genomic polyadenylation site deletion is followed by a 3′ gene with the same strand orientation and at least one intron, and where the splice sites involved are in sufficient proximity.

##### Change to Proximal Intronic Splice Motifs

We catalogued six cases where PEs were instigated by mutations within the same intron but beyond the PE splice motifs and branch points (Supplementary Figure S2C). Three of these mutations were 3′ of the PE (*FBOX38-*9-1, *MFGE8*-6-1, and *NPHP3*-3-1) and three were 5′ of the PE (*DMD-*56-1, *NR2E3-*7-2, and *RPGR*-9-1). Here we must clarify that although *DMD-*56-1 bore deletions both 5′ and 3′ of the PE, the reporting authors empirically demonstrated that only the 5′ deletion caused PE inclusion (Khelifi et al., 2011).

The *MFGE8-6* and *NPHP3-3* mutations were both SNVs similar distances from the PE donor sites (43 and 50 nt, respectively) that created new predicted FUS (hnRNP P2) binding motifs (Piva et al., 2012). Studies in mouse cells (Ishigaki et al., 2012) have shown that FUS binding along flanking introns can regulate alternative exon splicing in neuronal cells, so it is possible that a perturbation of normal FUS binding is responsible for these PEs escaping silencing.

A similarly positioned SNV in *FBXO38*-9-1 destroyed a predicted binding site for hnRNP K (Piva et al., 2012). This is consistent with a recent report demonstrating that hnRNP K depletion can lead to a widespread increase in cryptic exon inclusion, and that at least some of these cryptic exons are ordinarily silenced by hnRNP K binding within 100 nt of the 3′ intron (Bampton et al., 2021).

Unfortunately, there are few such similarities to connect the three PEs with 5´ distal mutations. The first, DMD-56-1, is caused by a 592nt deletion ending 26 nt 5´ of the PE acceptor site. The authors experimentally excluded modified branch point definition as a causative factor for this PE, but despite thorough experimentation with minigene assays they could not positively identify which components of the deleted region were responsible for the PE’s inclusion or what their mode of action was. The second PE with a distal 5´ mutation, NR2E3-7-2, was instigated by an SNV 581 nt upstream that altered multiple splice factor binding sites, making it difficult to predict which, if any, are mechanistically responsible. Incidentally, this was the same mutation that created an acceptor motif AG dinucleotide in NR2E3-7-1, though unlike the examples discussed in the first category of *Novel Pseudoexon Mutation Categories*, these two PEs exhibit mutually exclusive splicing.

In the third PE with a distal 5′ mutation, *RPGR*-9-1, a TTAAA motif is created 53 nt from the acceptor site. This motif is predicted to bind KHDRBS1 (Sam68) and/or KHDRBS3 (SLM-2), two splicing factors with high homology and similar effects on pre-mRNA splicing (Danilenko et al., 2017). In particular, KHDRBS1 has been shown to aid in the splicing of introns bearing *Alu* retrotransposon sequences (Pagliarini et al., 2020). Two such *Alu* elements occur within *RPGR* intron 9 (Supplementary Figure S3). Although the true pathology of this mutation is yet to be empirically determined, it may be that a disruption to KHDRBS1-mediated splicing is responsible for the *RPGR*-9-1 pathogenesis.

##### Unknown Mechanisms

In six cases, the connection between the identified mutation and the PE was unclear (Supplementary Figure S2D) and these cases bore no similarities to other catalogued examples. However, we note that *DYSF*-51-1b is an identical sequence inclusion to *DYSF*-51-1a (which arose from a PE donor site mutation), and has been observed at low levels in cells from healthy donors (Gonorazky et al., 2019); and similarly, that *DMD*-3-1a is also instigated by a 1 nt deletion in exon 5 (Supplementary Figure S1D).

### 3.5 Summary of Pseudoexon Mutation Analysis

Our pseudoexon catalogue, which is to date the most comprehensive ever assembled, confirms that PEs are most frequently instigated by direct mutation of their local splicing motifs; that the most frequently mutated components are the PE donor and acceptor splice motifs; and that the predominant type of instigating mutation is single nucleotide substitution. These findings support previously published observations of smaller pseudoexon datasets, which we gratefully acknowledge as secondary data sources for this catalogue. We add to this several novel classifications for rarer types of PE-instigating mutation, the most well-supported of these being mutations that weaken definition of adjacent canonical exons.

## 4 Latent Factors Contributing to Pseudoexon Splicing

Considering the complexity and stringency of vertebrate exon definition, in conjunction with the observation that single-nucleotide substitutions are the most frequent cause of PE pathogenesis, we are forced to question why these exon-like intron tracts exist in the first place. Even if the reference allele of a given PE is ultimately excluded from mature transcripts by the lack of one crucial splice motif, the presence of all the other exonic motifs might still encourage abortive “false start” activity by the spliceosome, wasting energy and unnecessarily prolonging mRNA maturation.

It may be that the latent elements of some PEs are mildly deleterious in this way but persist in the genome simply as another of evolution’s myriad compromises and works-in-progress. However, we must also examine the alternative explanation that these latent elements persist due to their spliceosome interactions being benign or even beneficial, and consider the various forms these interactions may take.

### 4.1 Canonical Exon Splice Variants

The earliest reported PE to meet the criteria of this catalogue (Dobkin et al., 1983) predates the completion of the first rough draft of the human genome project by nearly 18 years (Lander et al., 2001), and the years that intervened and followed these milestones have seen numerous revisions to the official coding sequences and transcript variants of thousands of genes. An inevitable side-effect of this progress is that many splicing phenomena initially reported as PEs have subsequently been reclassified as either canonical exons or mutant splice variants thereof. In the course of assembling and curating this catalogue, we separately collated 35 such examples (Supplementary Table S3). These examples could not be included in any of our PE analyses since there is no meaningful distinction between them and other canonical exon splice mutations. However, they serve as a useful reminder of the difficulty in distinguishing PEs from mutant variants of as-yet-unannotated canonical exons, especially if the canonical exons are expressed at low frequencies or in unexamined cell types (Ray et al., 2020). We expect that progress in transcriptomics will eventually necessitate similar reclassification for at least some of the PEs in this catalogue.

### 4.2 Novel or Unannotated Canonical Exons

Having excluded from of our catalogue those PEs that coincided with known canonical exons, we attempted to annotate additional examples of PEs that might undergo this reclassification in future. Our criteria for inclusion were 1) the PE must show evidence of splicing in normal cells for at least one of its splice sites, either in the original report, in non-cancer cell spliced expressed sequence tags (ESTs) from the UCSC Genome Browser’s “Spliced ESTs” track (Kent et al., 2002) or in paired-end RNAseq data (Sibley et al., 2015); and 2) inclusion of the PE in the mature transcript must be predicted not to trigger NMD.

A total of six PEs met these criteria (Supplementary Table S4). In all six cases, NMD avoidance was predicted due to preservation of the open reading frame and absence of any novel stop codons. We also allowed for cases where a transcript variant with a premature stop codon may have escaped NMD due the stop codon being introduced less than 55 nt from the final splice junction of the transcript (Zhang et al., 1998) but did not find any examples that met this criterion.

#### 4.3 Poison Exons and Decoy Exons

In recent years the term “poison exons” has been steadily gaining prominence in literature related to cryptic splicing phenomena. Carvill and Mefford (2020) characterised poison exons as conserved, alternatively spliced exons containing one or more premature termination codons that are spliced into unneeded transcripts to prevent their translation and target them for nonsense-mediated decay. “Decoy” exons behave similarly but are characterised by their additional capacity to non-productively interact with adjacent canonical splice sites, thereby promoting whole intron retention (Conboy, 2021).

There is a clear overlap between the definitions of poison/decoy exons and PEs, although the phenomena are not identical. Both describe non-canonical exon inclusions that generally impair the translation of full-length, functional protein from the affected transcript; but while PEs arise due to intragenic mutations and are often deleterious to the health of the patient, poison exons are a normal component of splicing that may contribute to fine-control of gene expression and are presumably beneficial, or at the very least benign.

Given the similarities between PEs and poison exons, and the relative novelty of the latter term, the intriguing possibility emerges that some of the splicing phenomena historically reported as PEs might be better re-classified as poison exons, or splice variants thereof.

Having already determined the concurring splice site reads between our PE catalogue, and ESTs and RNAseq data (see Section 4.1) we separately tabulated all those examples where evidence supported their splicing in normal cells, but which did not preserve the transcript open reading frame (Supplementary Table S5).

A possible reason for the high number of candidate poison exons seen in *NF1* and *DMD* is the exceptional size and high intron count of these genes. These features unavoidably entail a long transcription and maturation time, which must be reconciled with the fact that the quantity of any encoded protein that the cell needs can change dramatically in a matter of seconds. The more poison exons a transcript contains, the more possible time-points there are for interrupting the reading frame and preventing an unneeded transcript from reaching functional maturation.

Of the 413 catalogued PEs, for 65 (15.7%) we found evidence of splicing of at least one splice site in normal cells. This is a remarkably high concordance when one considers that, for the most part, splicing of putative PEs in normal cells is not something that has been systematically investigated; as such, what supporting evidence there is exists largely by chance. As RNAseq becomes more commonplace and is applied with greater sensitivity and read depth to a broader range of cell types, it may emerge that many more PEs—perhaps even a majority—originated as benign rare exons or functional exon-like intronic sites.

#### 4.4 Recursive Splice Sites

In a previous report focused on PEs in the *DMD* gene (Keegan 2020), we examined the possibility that some PEs may arise from the errant splicing of predicted recursive splice sites (RSSes). Here we sought to examine this possibility as it applies to our total set of PEs. This task was complicated by the fact that there is as yet no consensus on the precise definition of RSSes and how best to experimentally verify their presence. For example, the criteria employed by Zhang et al. (2018) required that a putative RS-exon should bear an agGT tetranucleotide at the acceptor site and that the nucleotides around the acceptor site should be highly conserved, while the approach of Wan et al. (2021) was agnostic to sequence conservation.

We searched the splice site coordinates of our PE dataset against five published datasets of recursive splice sets (Kelly et al., 2015; Sibley et al., 2015; Blázquez et al., 2018; Zhang et al., 2018; Wan et al., 2021). We did not find any matches in Kelly et al. (2015) or in the filtered results of Sibley et al. (2015), but we did discover seven matches in the filtered results of the other three reports—five in Wan et al. (2021) and one in each of Blázquez et al. (2018) and Zhang et al. (2018) (Table 2). To our knowledge, this is the first conclusive evidence supporting our earlier hypothesis that pathogenic PEs can arise from mutations near recursive splice sites (Keegan 2020). Additionally, we were interested to note that six of the seven recursive splice sites were also spliced as components of putative poison exons in normal cells (Supplementary Table S5), with *COL4A5-*6-1 being the exception. This may indicate that these sites serve a dual purpose in splicing regulation, though this remains to be confirmed through functional studies.

**TABLE 2:** Pseudoexons associated with seven confirmed intronic recursive splice sites.

Gene	Intron	#	Start	End	Size	Pseudoexon mutation(s)	ME-A	ME-D	PE RNA source	PE references	RSS RNA source	RSS references
*ATM* (NM_001351834.2)	27	1b	108287410	108287521	112	c.3994-159A>G (A+32)	7.71 -> 8.12	8.49	LCLs	Coutinho et al. (2005)	HBECs	Wan et al. (2021)
		1a		**108287438**	29	c.3994-193C>T (A−3)		**6.38**	LCLs; peripheral blood	Coutinho et al. (2005), Královičová et al. (2016), Landrith et al. (2020)		
*COL4A5* (NM_000495.5)	6	1	108570649	**108570795**	147	c.385-719G>A (A+46)	5.25	**7.51**	Hair bulb	King et al. (2002)	HBECs	Wan et al. (2021)
*FBOX38* (NM_030793.5)	9	1	**148411080**	148411238	159	c.1093+532C>G (D+59)	**9.11**	6.57	Whole blood, lung tissue	Saferali et al. (2019)	HBECs	Wan et al. (2021)
*GLA* (NM_000169.3)	3	1	101401233	**101401347**	115	c.547+395G>C (D−5)	**5.10**	7.82	Whole blood	Higuchi et al. (2016)	Cerebellum, K562 cells	Blázquez et al. (2018)
*MCCC2* (NM_022132.5)	10	1	**71636104**	71636167	64	c.1054G>A (e11 D-19)	**5.72**	3.24	Emetine-treated fibroblasts	Stucki et al. (2009)	HBECs	Wan et al. (2021)
*NPHP3* (NM_153240.5)	3	1	**132717955**	132718117	163	c.671-996C>G (D+50)	6.50	**0.56**	Leukocytes	Larrue et al. (2020)	HBECs	Wan et al. (2021)
*OCRL* (NM_000276.4)	4	1	**129553236**	129553301	66	c.239-4023A>G (D+1)	**8.18**	2.68 -> 10.86	Skin fibroblasts	Rendu et al. (2017)	PA1 cells	Zhang et al. (2018)

Genomic coordinates of the recursive splice sites and their maximum entropy scores are in bold text. “ME-A” and “ME-D” refer to the Maximum Entropy scores for the acceptor and donor splice sites, respectively.

### 5 Unique Cases and Additional Observations

#### 5.1 No Known Pseudoexons are Processed by the U12 Spliceosome

The minor spliceosome, or U12 spliceosome, processes just 0.37% of all human introns (Olthof et al., 2019). Type-U12 introns can most easily be recognised by their highly conserved donor-site (UTATCCT) and branch point (CCTTUAY) motifs, and their tolerance for AT-AC terminal dinucleotides—although the latter feature is not present in all U12 introns and GT-AC, AT-AG or GT-AG terminal dinucleotide pairs are also observed (Turunen et al., 2013).

A search of the donor sites of all catalogued PEs and their 5′ spliced exons discovered a single example of a UTATCCT donor site motif, at the donor site of *LHCGR*-6-1b. Although this donor site scores low as a U2 splice site (MaxEnt = 0.48), there was no type-U12 CCTTUAY branch-point motif near the acceptor site of the 3′ exon 7, and the canonical 5′ exon 6 did not have a type-U12 donor site. This indicates that the termini of *LHCGR* intron 6 have evolved to be removed via the predominant mode of U2 splicing. It therefore appears that this PE is spliced via the U2-spliceosome and not the U12. Therefore, we concluded that no U12-spliced PEs are reported in this dataset, although we did note that *STK11*-1-1 occurs in a U2 intron that is 5′ adjacent to a known U12 intron (Hastings et al., 2005).

While a type-U12 PE may yet be reported, it is unsurprising that none have been discovered thus far. The great majority of reported PEs have been observed as singletons that are directly spliced to canonical upstream and downstream exons in the mature transcript. This means that each of the two PE splicing reactions involves one neighbouring canonical splice motif that has evolved for optimal interaction with a particular spliceosome. From this we can infer that the mode of a PE’s splicing will largely be determined by the splicing mode of its encompassing intron, i.e., a U2-spliced PE cannot arise within a U12 intron or vice versa. A similar hypothesis was suggested by Qu et al. (2017) in their analysis of U12 splice mutations. Because only 0.37% of human introns are type-U12 (Olthof et al., 2019) the genomic range within which a U12 PE could plausibly arise is vanishingly small. However, exceptions may occur if a mutation that prevents proper recognition of the splice motifs of a U12 intron results in cryptic U2 splicing. Madan et al. (2015) observed such cryptic U2 splicing of a U12 intron of *WDR41*, though this was the result of knockdown of the splice factor ZRSR2, rather than mutations in *WDR41* itself.

#### 5.2 Pseudoexons With Non-AG Acceptor Sites Occur Rarely but Unpredictably

We catalogued four examples of PE variants with non-AG acceptor site dinucleotides. Three of these (*RB1-*14-1b, *RB1-*14-1c, and *RB1-*14-1d) arose from the 5′ junction of a single LINE-1 retrotransposon insertion in *RB1* intron 14 (Rodríguez-Martín et al., 2016). These three variants share a common U2-type donor site, but each have unique non-canonical acceptor sites that were confirmed through Sanger sequencing. This LINE-1 insertion also induced an additional PE variant with a canonical acceptor site (*RB1-*14-1a).

The fourth non-canonical PE (*NF1*-39-1a) was observed in *NF1* as the result of a donor-site-creating SNV. Like the *RB1* PEs, *NF1*-39-1a bears a canonical donor site and a non-canonical acceptor site and shares its donor site with a wholly canonical variant, *NF1*-39-1b.

A report by Parada et al. (2014), examined common features of 184 non-canonical splice sites, and the authors observed therein that the terminal dinucleotides of most non-canonical splice sites differ from the canonical AG or GY pairs by only a single nucleotide. This holds true for the *RB1* non-canonical PEs, which have CG, AT and AT respectively as their acceptor-site terminal dinucleotides, and for *NF1*-39-1a, which has a TG dinucleotide. We speculate that this one-nucleotide rule is observed because varying only a single nucleotide minimises the amount of resistance that must be overcome to “persuade” the spliceosome to cleave at a non-AG/GY dinucleotide.

Unfortunately, there are few other established hallmarks for human non-canonical exons that these PEs can be compared against. Burset et al. (2000) suggested that non-canonical splice sites may parasitically exploit the presence of nearby canonical splice motifs to recruit the spliceosome, an hypothesis supported by the alternative canonical acceptor sites observed in *RB1-*14-1a and *NF1*-39-1b. However, even if this “parasite” model accounts for spliceosome recruitment, it still begs the question of why the non-canonical splice sites are used at all when workable canonical sites are available. Similarly, although Parada et al. (2014) detected a higher density of ESEs and intronic splice enhancers around non-canonical sites, it is not valid to apply their statistical analysis to just four additional sites. Deriving a complete explanation for why these two mutations in *RB1* and *NF1* created PEs with non-canonical splice sites, when so many other similar mutations in these and other genes did not, therefore remains as a challenge for future researchers.

#### 5.3 Terminal Pseudoexons are Both Rare and Difficult to Detect Without Third-Generation Sequencing Technologies

**FIGURE 3:**
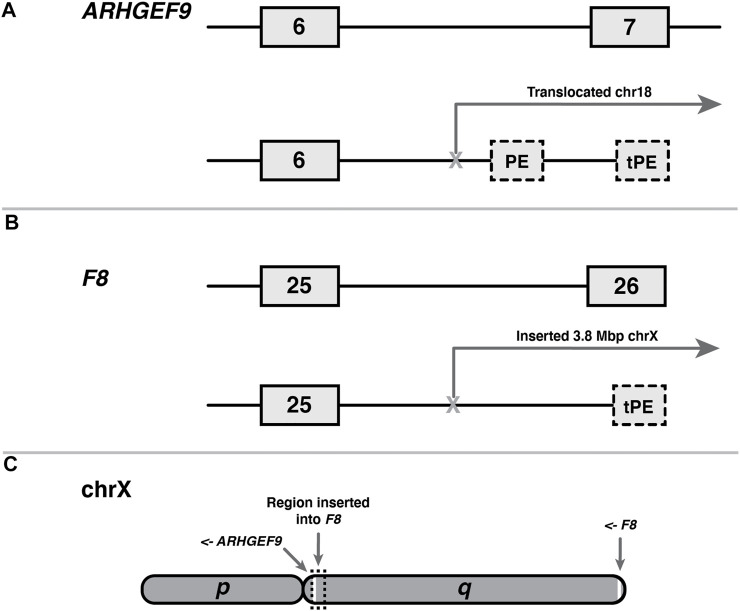
Shared features of two terminal pseudoexons (tPEs). **(A)** *ARHGEF9* tPE (*ARHGEF9*-6-2) and internal PE (ARHGEF9-6-1) arising from within a translocated region of chromosome 18. **(B)** F8 tPE (F8-25-1) arising within intragenic sequence of a transposed 3.8 Mb tract of the X chromosome. **(C)** Relative locations, to scale, of the affected genes on the X chromosome q-arm, and genomic origin of transposition in patient B. Arrows indicate reading directions of each gene.

We catalogued two examples of terminal pseudoexons (tPEs), each arising from unique mutations in *ARHGEF9* (Figure 3A) and *F8* (Figure 3B). Although it is difficult to generalise from just two observations, there are obvious similarities between these cases that are worth noting. Both the *ARGHEF9* and *F8* genes are carried on the *q* arm of the X-chromosome, albeit at opposite ends (Figure 3C), and in both cases the instigating mutations entail large sequence rearrangements that moved the canonical 3′ end of the gene out of splicing range of the upstream exons. In the case of *ARHGEF-*6-2, this mutation is a balanced crossover with chromosome 18, while the *F8* gene of the second patient bears a 3.8 Mb insertion of chromosome X intergenic sequence. Notably, the region inserted into *F8* in *F8-*25-1 encompasses *ARHGEF9* along with 11 other protein-coding genes (not shown). Interestingly, although the *ARHGEF9* mutation was originally described as creating two tPEs—one 5′ of the breakpoint in the normal intron 6 sequence, and one in the translocated chromosome 18 sequence—we found that the first of these terminal exons shares its polyadenylation site with *ARHGEF-IT1*, a noncoding and largely uncharacterised two-exon transcript nested within *ARHGEF* intron 6. Because it shares sequence with a canonical exon, this mutant terminal exon therefore does not meet the criteria for classification as a tPE.

The fact that tPEs are so rare in comparison to internal PEs is surprising when one considers that the defining hallmarks of a last exon are comparably well-defined to those of an internal exon, and therefore they should be expected to arise from random mutation at roughly the same frequency. The requirement for a functional acceptor site is similar in both exon types, and the requirements for polyadenylation site definition (Kaida, 2016) do not appear very much stricter than those for donor site definition. Furthermore, last exons usually contain a stop codon, a requirement that most PEs meet by default, and last exons also exhibit a much broader range of sizes than internal exons (Movassat et al., 2019).

We suggest that there are at least three compounding causes for the low discovery rate of tPEs. The first is that the laboratory techniques required to confirm a tPE make them considerably more difficult to discover than internal PEs. Many internal PEs were detected serendipitously when researchers noticed unusually large products from their RT-PCRs of flanking canonical exons, but this method of discovery is only possible if the RT-PCR primer target sites are present on both sides of the mRNA insertion, and this is not the case for tPEs as they are not spliced to any 3′ exon. Any RT-PCR of the canonical exons flanking a tPCR would produce either low abundance products of the expected size (if some level of normal splicing is still present) or no products at all. Even if the researcher eventually discovers the acceptor splice site of the tPE, their subsequent failure to detect an active donor site may lead them to conclude that the effect of the mutation is partial intron inclusion and arrest of splicing.

A third possible contributing factor is that terminal exon definition may be a stricter process than it appears. At the very least, the similarity of the two mutations described here suggests that the absence of competition from downstream canonical exons is a contributing factor, which is something that can only occur after large-scale sequence rearrangements such as these. Conversely, the effect of an *EPCAM* terminal exon deletion mutation (Supplementary Figure S2B) was to induce a fusion transcript with *MSH2* but no novel polyadenylation site, as in this case a latent intergenic pseudoexon combined with the chromosomal proximity of *MSH2* provided viable splicing partners.

Regardless of the true frequency of tPEs, it is worth noting that the aforementioned barriers to their detection do not apply to third generation sequencing technologies like Nanopore, which are largely agnostic in their detection of polyadenylated transcripts. As the uptake of Nanopore and other third-generation RNA sequencing technologies continues to increase, there may be a corresponding increase in the discovery rate of tPEs.

### 6 Conclusion

Pathogenic pseudoexons primarily arise from mutations that directly enhance their donor or acceptor site motifs. However, other types of instigating mutation are also observed less frequently, but with consistent features, many of which are characterised for the first time in this report. In rare cases, the splicing pathology of a PE was highly idiosyncratic and could not be properly categorised due to a lack of similar supporting examples. These findings advance our understanding of how mutations give rise to pathogenic pseudoexons, but also highlight that our understanding is still far from complete.

We also discovered seven examples of pseudoexons that coincide with recently confirmed recursive splice sites, conclusively demonstrating that functional exon-like intron elements can be converted to pseudoexons when favourable mutations arise nearby. Although it remains to be determined how many pseudoexons arise in this way, we found that 15.7% of pseudoexons showed evidence of splicing at one or both of their splice sites in cells from healthy donors, a figure that is likely to increase further as the fidelity and quantity of RNAseq data continues to improve.

## Data Availability

The original contributions presented in the study are included in the article/Supplementary Material, further inquiries can be directed to the corresponding author.

## Supplementary Material

Supplementary Figure 1 Pseudoexons instigated by weakened definition of nearby canonical exons. Pseudoexons are shown as medium grey dash-line boxes, with flanking canonical exons in light grey and intervening introns as solid black lines. Sizes are not to scale. Dash-lines elsewhere indicate altered splicing of canonical exons. Roman numerals within pseudoexons (I–IV) indicate identical sequence inclusions (see also Supplementary Figure S2). Selected relevant features for each mutation are indicated where appropriate, with full details and references provided in Supplementary Table S1. Numbers above exon ends indicate acceptor and donor Maximum Entropy splice site scores, with “NF” indicating a site that is non-functional in the reference (NF -> score) or mutant (score -> NF) allele due to lacking an essential AG-GY dinucleotide. Changes to exon HExoSplice scores are shown above the affected exons as “∆Hx,” other motif changes are indicated with vertical lines and labelled as “± name-of-motif.” Vertical brackets indicate common features of enclosed splice events. Note that *GAA*-1-1 is shown twice due to the splicing effects of its mutation fitting both the **(A,B)** sub-categories.

Supplementary Figure 2 Pseudoexons instigated by novel or unknown mechanisms. Pseudoexons are shown as medium grey dash-line boxes, with flanking canonical exons in light grey and intervening introns as solid black lines. Sizes are not to scale. Dash-lines elsewhere indicate altered splicing of canonical exons. Roman numeral “IV” indicates an identical sequence inclusion to one shown in Supplementary Figure S1. Selected relevant features for each mutation are indicated where appropriate, with full details and references provided in Supplementary Table S1. Numbers above exon ends indicate acceptor and donor Maximum Entropy splice site scores, with “NF” indicating a site that is non-functional in the reference (NF -> score) or mutant (score -> NF) allele due to lacking an essential AG-GY dinucleotide. “+D” indicates one or more gain-of-donor-motif mutations. Other motif changes are indicated with vertical lines and labelled as “± name-of-motif.” Vertical brackets indicate common features of enclosed splice events. *Variant-induced increase in splicing of *FBXO38*-9-1 was low (from 8 to 13% of *FBXO38* transcripts) but statistically significant (Saferali et al., 2019).

Supplementary Figure 3 A G>A substitution within *RPGR* intron 9 creates a binding motif for KHDRSB1 (Sam68), potentially altering the splicing of an adjacent pseudoexon (*RPGR*-9-1) that originates within an *AluSx* element. Image captured from *UCSC Genome Browser*, GRCh38/hg38. Location of TTAAA motif indicated with a thick vertical red line, and additional annotations are written in red text. Scale at top shows chromosome X coordinates. “GENCODE V36” track shows aligned transcripts, with gene names on the left, solid bars indicating exons and horizontal lines indicating introns. Arrows (< and >) on introns indicate transcript reading direction relative to chromosome + strand. “Repeating Elements” track shows alignments of SINE or LINE elements as solid rectangles, with darker shading indicating higher homology to the reference sequence for that element.
