# Corrigendum: Analysis of Pathogenic Pseudoexons Reveals Novel Mechanisms Driving Cryptic Splicing

**DOI:** 10.3389/fgene.2022.943044

**Published:** 2022-06-09

**Authors:** Niall P. Keegan, Steve D. Wilton, Sue Fletcher

**Affiliations:** ^1^ Centre for Molecular Medicine and Innovative Therapeutics, Health Futures Institute, Murdoch University, Perth, WA, Australia; ^2^ Centre for Neuromuscular and Neurological Disorders, Perron Institute for Neurological and Translational Science, The University of Western Australia, Perth, WA, Australia

**Keywords:** pseudoexons, cryptic splicing, splicing mutations, recursive splicing, poison exons, genetic disease

In the original article, there was a mistake in the Supplementary Tables file as published. An incorrect version of the Supplementary Tables file was uploaded, resulting in Supplementary Table S3 being absent and Supplementary Tables S4, S5 being mislabeled. The corrected Supplementary Tables file has been added in Supplementary Material.

The authors apologize for this error and state that this does not change the scientific conclusions of the article in any way. The original article has been updated.

