# Supplementary figures and images for: Analysis of Pathogenic Pseudoexons Reveals Novel Mechanisms Driving Cryptic Splicing
Source: Front Genet. 2022 Jan 24;12:806946. doi: 10.3389/fgene.2021.806946 (PMC8819188; doi:10.3389/fgene.2021.806946)

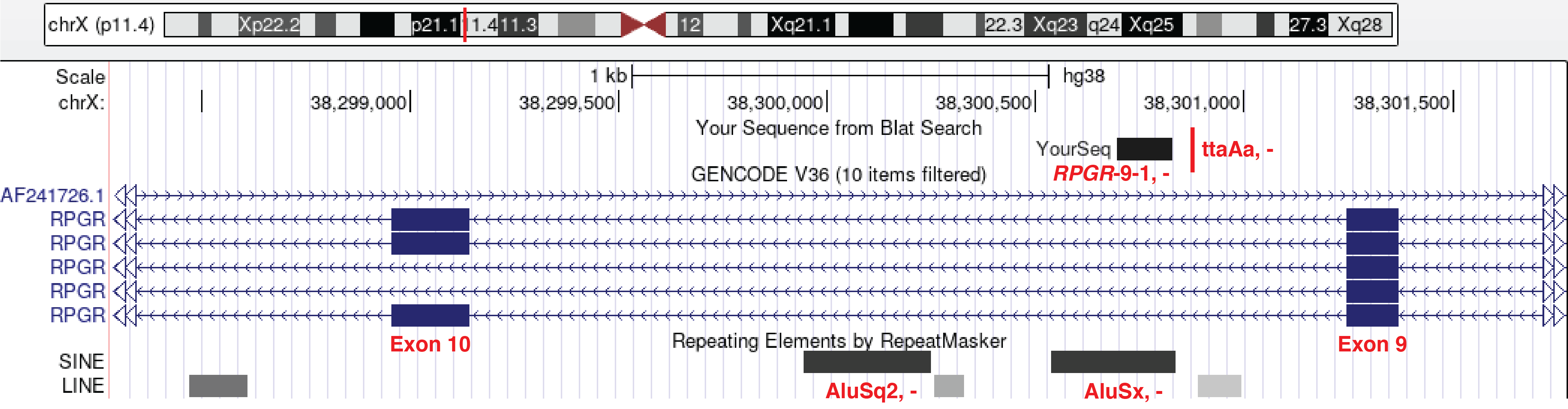

Supplement: Supplementary file 1 [file Image3.TIF]

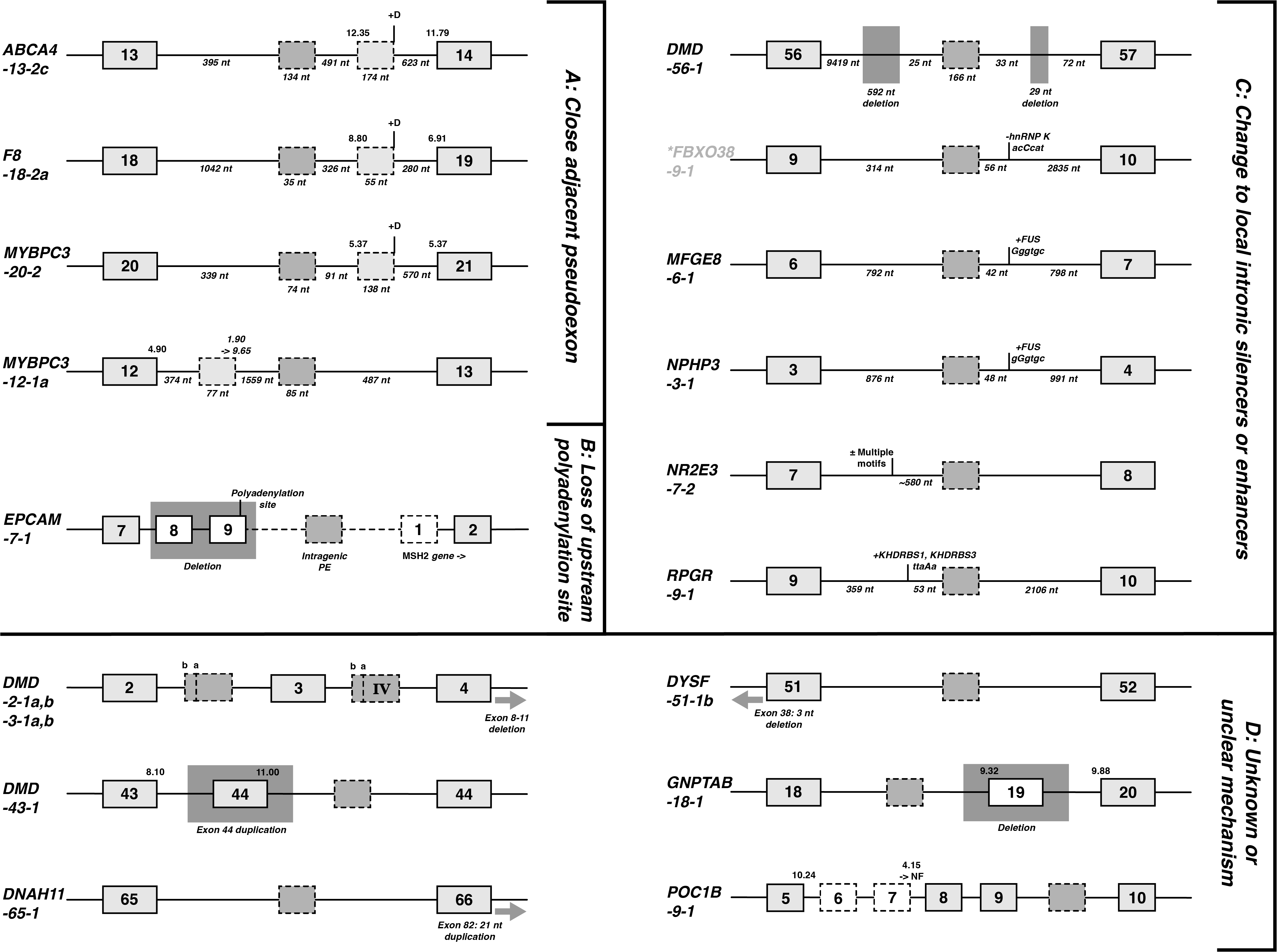

Supplement: Supplementary file 2 [file Image2.tif]
